# Torsion—Resistant Structures: A Nature Addressed Solution

**DOI:** 10.3390/ma14185368

**Published:** 2021-09-17

**Authors:** Federica Buccino, Giada Martinoia, Laura Maria Vergani

**Affiliations:** Department of Mechanical Engineering (DMEC), Politecnico di Milano, Via La Masa 1, 20156 Milano, Italy; federica.buccino@polimi.it (F.B.); giada.martinoia@mail.polimi.it (G.M.)

**Keywords:** torsional failures, torsional resistance, process model, bio-inspired structures, metamaterials

## Abstract

The complexity of torsional load, its three-dimensional nature, its combination with other stresses, and its disruptive impact make torsional failure prevention an ambitious goal. However, even if the problem has been addressed for decades, a deep and organized treatment is still lacking in the actual research landscape. For this reason, this review aims at presenting a methodical approach to address torsional issues starting from a punctual problem definition. Accidents and breaks due to torsion, which often occur in different engineering fields such as mechanical, biomedical, and civil industry are considered and critically compared. More in depth, the limitations of common-designed torsion-resistant structures (i.e., high complexity and increased weight) are highlighted, and emerge as a crucial point for a deeper nature-driven analysis of novel solutions. In this context, an accurate screening of torsion-resistant bio-inspired unit cells is presented, taking inspiration specifically from plants, that are often subjected to the torsional effect of winds. As future insights, the actual state of technology suggests an innovative transposition to the industry: these unit cells could be prominently implied to develop novel metamaterials that could be able to address the torsional issue with a multi-scale and tailored arrangement.

## 1. Introduction: The Destructive Power of Torsional Load and the Extended Interest in It

Failure due to torsional loads is a challenging and impactful issue to be addressed: it is commonly present in several fields and it has been observed and analyzed for more than two centuries.

Many authors have offered analysis methods for components subjected to torsion, which involves the use of approximations and simplifications due to the high complexity of the problem, characterized by a three-dimensional nature and seldom observed as purely applied, since it is often combined with bending, compression, and tension loads.

Furthermore, different approaches to find torsion-resistance solutions can be used. As an example, the problem could be faced through a classical mechanical analytical approach, as done by De Saint-Venant [1] in 1855, who implemented the analysis of a solid linear elastic homogeneous isotropic beam subjected to torsion. In the last century, different types of approaches have been exploited. In the first decades of the 1900s, the development of new technologies and components led to the exploitation of experimental analysis to face the torsional problem [2,3]. Indeed, for structures with complex geometries and heterogeneous materials, it was extremely difficult to define an exact mathematical model and to calculate its accurate solution. The limitation of these experimental approaches, however, was the low level of accuracy provided by the experimental equipment of those years [2]. In the 1960s, numerical methods, which exploit the application of a mathematical model capable of providing an approximation of the problem, have been implemented and used [4], overcoming the practical limitations of the testing apparatus. In the meantime, both the results of experimental and numerical analysis and the implementation of more complex mathematical models has allowed to confirm and further explore the proposed theoretical models [5], which have been refined and implemented for different types of components’ geometries [6] and complex materials such as concrete [7,8].

Starting from the 1980s, a disruptive new approach to solve the torsional problem has been proposed and includes the understanding and search of torsion-resistant natural structure strategies, thus engaging bio-inspiration [9,10,11,12,13,14].

In later years, even complex torsion-related problems such as non-linear analyses [15,16,17,18,19], dynamic analyses (for structures subjected to vibration or to fatigue) [20,21,22,23,24,25,26,27], and analysis of shell [28,29,30] and frame [31] structures have been addressed through analytical and numerical methods. Recently, solutions to the torsional issue focus on the development of novel meta-materials [32,33,34], which can also be bioinspired.

Despite the high variety of strategies used, this wide problem has not been fully and systematically approached yet, even though torsional load must be taken into consideration in the design of most components, whether being shafts or far more complex structures as in the case of ships, buildings, and aircrafts.

**Figure 1:**
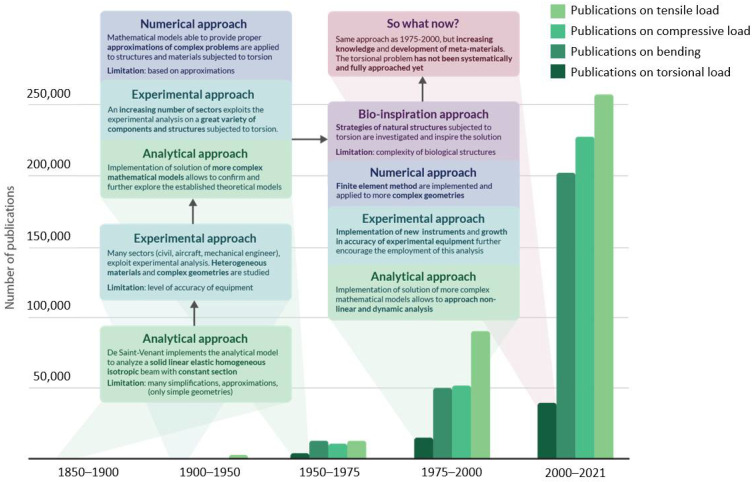
The infographic presents the evolution in approaching the torsional problem across years and the occurrence of publications dedicated to torsional analysis from 1850 until today. For each period, the comparison of torsional works with the papers dedicated to other commonly applied loads (tension, compression, and bending) is reported. The green boxes focus on the evolution of the analytical approach and their limitations; the light-blue boxes refer to issues related to the experimental approach. Violet boxes are dedicated to observations concerning the numerical approach, while the purple rectangle concerns bio-inspiration strategies. In pink, the latest trends in addressing the torsional problem are reported.

The evolution in approaching the torsional problem across years is presented in Figure 1, which reports the occurrence of publications dedicated to torsional analysis and a comparison with works dedicated to other more commonly applied loads such as tension, compression, and bending. It is immediately evident that the research devoted to the torsional problem is drastically limited in comparison with the more common loads, specifically because of the already mentioned reasons.

The first point in order to comprehend and deal with the multi-faced and challenging aspects of this topic is to deeply analyze failure issues generated by torsion, being a pure or key contribution to failure.

### 1.1. Failure Due to Torsional Load

**Figure 2:**
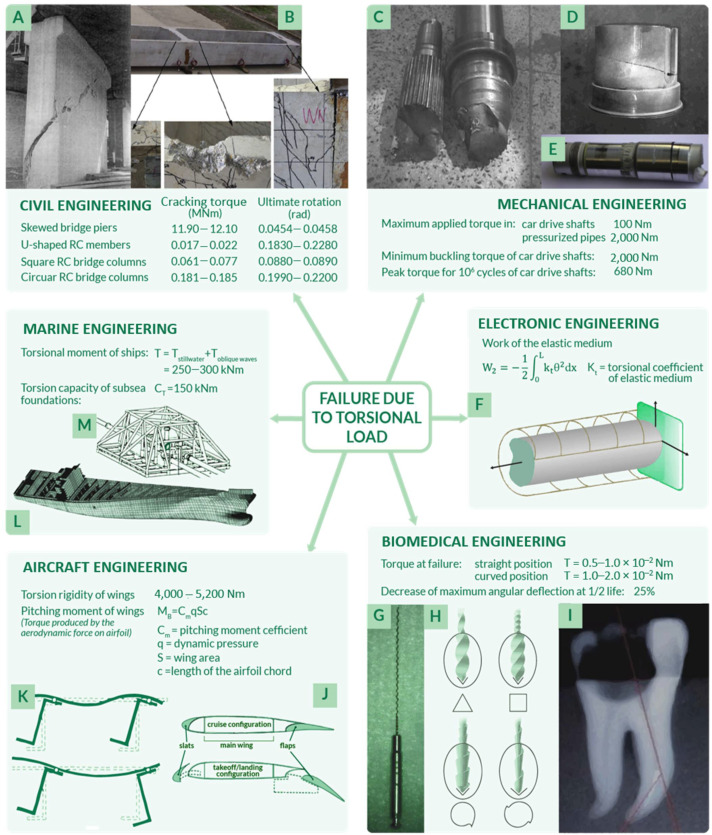
Failure due to torsional load affect many fields of interest such as civil (**A**,**B**), mechanical (**C**–**E**), electronic (**F**), biomedical (**G**–**I**), aircraft (**J**,**K**), and marine (**L**,**M**) engineering. For each sector, specific torsional-related failures are reported.

As above-mentioned, several fields are affected by torsional damage and even failure; a detailed analysis of sector-specific torsion-related issues is presented in this paragraph and schematized in Figure 2.

An area of interest in which torsion is a matter of concern is **civil engineering**, where the aim is to better comprehend the behavior of buildings, bridges, or simply concrete beams under torsional static loads or dynamic loads, as in case of seismic events. In 1969, the theory of torsion in this sector has been described in more detail by Koll-Brunner and Basler [5], focusing on methods for the analysis of torsion of single-span or continuous members through the use of familiar tools for structural engineers. These solid, thin-walled, open or closed cross section structures are base elements for constructions, which even actually embrace torsion-related failures. For instance, as explained in the research of Kawashima et al. [18], piers of a skewed bridge could fail if subjected to extensive torsion damage (shown in Figure 2A) as a consequence of an earthquake. The damage was a direct effect of seismic activity and it could be clearly recognized that the torsional fatigue failure crack had a 45° inclination.

To be more precise, the load generated on bridge columns, foundations, walls, and in other civil structures is never steady and pure torsion, but usually a combination of different types of applied loads [16,35], characterized by a cyclic nature as analyzed by Kelly et al. [36]. Indeed, reinforced concrete beams, which undergo torsion failure, are a matter of concern even if strengthened with FRPs (fiber reinforced polymers), specifically designed for constructions. However, research on this topic is extremely limited [16], even though many authors have focused their research on beams under pure torsion condition, and specifically on both open section beams as in U-shaped thin-walled analyzed by Chen et al. [37] (Figure 2B), and closed section beams such as those described by Chariolis [38], Rao et al. [39], and Mondal et al. [16]. Again, in Figure 2B, the typical inclination of 45° of torsional fatigue failure cracks could be observed. Note that the first attempts in investigating concrete beams under torsion loads go back to 1900 [2]: in the first fifty years of the twentieth century, many suggestions to determine a reliable analytical criterion and methodology for concrete beams subjected to combined stress due to bending and torsion were proposed [2,7]. As reported by Fisher [2], who performed both experimental tests on cylindrical reinforced concrete beams, a suitable failure criterion in these conditions could be maximum stress theory. As further reported by Kemp et al. in 1971 [7], the major issue related to reinforced concrete subjected to torsional load is that the applied loading condition is neither homogeneous nor isotropic. This leads to a lack of mathematical rigor and thus uncertainty in the design phase. Another possible reason for the lack of studies on torsion in civil structures relies on the fact that buildings are usually assumed to be composed of articulated simple vertical or horizontal elements specifically arranged so that torsion could be eliminated in the structural analysis. In case it could not be completely neglected, torsion is usually included in the safety factor choice in the design phase. Not only methods to prevent torsion failure, but torsion failure mechanisms have also been analyzed with the aim to better recognize and characterize them. Indeed, as previously anticipated, one of the main issues related to torsion analysis is that it is difficult to isolate, recognize, and observe, usually combined with other types of load (i.e., bending [2,6,8]). These usually have shell geometries, characterized by a far more complex analysis of stresses and strains if compared to beams [5]. To approach this complexity, many authors have suggested tailored torsion analysis methods, as in the case of Kumari et al. [29], who analyzed the behavior of a conoidal shell. Similarly, Zheleznov et al. [17] focused their attention on the issues of the stability of elliptical cylindrical shells subjected to torsion and internal pressure and solve them from the analytical point of view in the case of nonlinear deformation.

Another field of interest is **mechanical engineering**, in which failures of shafts due to transmission of torque moments are frequently observed. Power transmission shafts are fundamental components of engines, turbines, and gearboxes, where torque resistance of shafts must be assured in both static and fatigue loading conditions [40]. In this regard, there are many fragmented analyses of solid and hollow shafts that failed due to torsional fatigue [41,42,43,44]. It has been observed that the effects of torsional fluctuating stress lead to unexpected failure, which shortens the predicted usage life: shafts are usually subjected to both torsion and bending loads, so failure may occur either at the maximum bending or torsional point. Considering the specific case of a crankshaft, failure occurs due to high stresses in a specific position along the component as a combination of maximal engine torque and maximal bending stress. Another remarkable fact is that cracks present in shafts that fail due to the combination of torsion and bending, as in the case of failed transmission shafts shown in Figure 2C–E, have the inclination of torsional fatigue failure cracks in brittle materials observed through visual macroscopic analysis, corresponding to 45°, as in the mentioned case of concrete beam failure cracks.

Thus, taking into consideration the failure due to torsional load in shafts, similar considerations could be extended to other sectors such as automotive [45,46,47,48,49], aerospace [43,45,50,51,52,53], agriculture [54,55,56], and energy production industry [57].

In the previous failure examples, the structures subjected to torsion were beams, with solid or hollow section, and shells. For the sake of completeness, it should be taken into consideration that the components’ behavior also strongly depends on their size and geometry. For instance, in the field of **electronic engineering**, rods with micro and nano dimensions present in micro-sensors and actuators are typically subjected to torsional vibrations [20,21] (Figure 2F). This dynamic condition could result in failure for rods with different shapes and sizes. This specific issue was investigated by Hassannejad et al. [21], who managed to prove the influence of geometry on the rods’ behavior under vibrational torsional loads.

Another peculiar example is related to the **biomedical field**, in which rotary endodontic instruments are commonly used. They have a very complex geometry and are highly subjected to torsional loads [58,59,60]. These hand-operated instruments are characterized by reduced dimensions, from hundreds of microns up to a millimeter [61], and have different cross-sectional shapes, as illustrated in Figure 2G,H [62], according to the specific function they are designed for. Improving torsion strength and torsion fatigue resistance in all these instruments could prevent failure, avoiding dangerous and complicated operations related to their unneeded extraction from teeth [63,64,65] (Figure 2I).

Another important field of interest is **aircraft engineering**, in which some shell and frame structures are subjected to torsional stresses: indeed, both fuselage [3,66,67,68,69] and wings [53,70,71] must fulfil geometric constraints to be as light as possible and face bending and torsional loads. To withstand these loads during take-off, flight, and landing, avoiding fatigue or static failure, these components must be specifically designed, with ad hoc features that guarantee increased stiffness and structural integrity. Considering wings, particular attention should be given to the actuation of aircraft flaps and slats (Figure 2J), which are strongly subjected to torsional loads during the flight phase [70]. In the case of fuselage panels, the main issue concerns the different possible deformation modes due to flexure–torsion, as shown in Figure 2K.

Additionally, even **marine industry** researchers have focused their attention on torsion, trying to identify the effect of shear stresses in thin-walled ships to avoid failure. Indeed, ship hulls [72] and ultra large container ships [73] are subjected to significant torsional moments, up to 300 kN∙m, due to both an improper distribution of cargo loading and fuel, and the presence of massive oblique waves (Figure 2L) [74]. In the case of reduced torsional stiffness, torsional loads could lead to failure of the ship, which could consequently cause environmental disasters as well explained by Shama [74] in his work, almost entirely dedicated to torsional load effects in ships. Moreover, frame, shell, and beam structures placed underneath the sea are also interested by torsional loads, as in the case of subsea foundations and piles or mooring applications analyzed by different researchers [31,75,76]. Considering the example of shallow foundations, a proper case study is the interaction between torsional and sliding loads, which might not be supported by structures such as oil pipeline end manifold and pipeline end termination systems (Figure 2M), as studied by McDonald et al. [31].

The impactful failures due to torsional load that have affected several engineering fields require a systematic discussion, starting from a comprehension of the complex torsional-related problem.

### 1.2. Torsion-Resistance: A Complex and Underestimated Issue

Torsional load effects have been preliminarily analytically considered since the 1850s, focusing on cylindrical elastic corpses described in the theory of Saint-Venant in “De la torsion des prismes” [77]. It has been known long before this theory that torsion is characterized by some peculiarities, which make its analysis very complex and challenging, even if this load is applied to cylindrical beams. Indeed, torsional load is not axial-symmetric and this means that it has a three-dimensional nature. For this reason, it is not possible to analyze torsion effects on components or design a torsion resistant structure without working in a complex three-dimensional space. According to this, in the case of components with more intricate geometries than cylindrical beams, the analysis of stresses and deformations is articulated to perform, as proven by the analysis and computational models of several researchers [4,5,25,74,78,79,80,81,82]. To sum up, considering a torque applied on structures with complex geometries such as those made of different interacting beams or thin walls, as in the case of the mentioned skirted foundations, the mechanical analysis of stresses and strains requires high computational cost and needs detailed meshing strategies.

Since the design of a component or a structure must pass through the optimization step, the complexity of calculating stresses and strains occurring due to the applied torsional loads is currently a limit. For instance, considering the case in which a lightweight structure is specifically required, it is not always possible to optimize the weight observing the structural constraints such as keeping the torsional rigidity constant [83].

This difficulty must be added to another issue related to torsion analysis, concerning the fact that this type of load is rarely the primary cause of failure: it is often combined with bending [83] and it frequently does not directly cause failure, even though it contributes to it. One of the actual challenges is to determine the contribution of torsion and to associate its effects in terms of strains and deformations.

Another reason that could explain the lack of research progresses in the optimization of torsional strength might be the complexity of analysis in the case of structures with very low or large size. On one hand, low size components, as in the case of rotary instruments, are difficult to observe and monitor under the application of load. On the other hand, structures such as aircraft wings and fuselages, buildings, bridges, ultra large container ships, and wind turbines are also difficult to analyze due to the presence of many multi-axial stress conditions.

## 2. Common-Designed Torsion-Resistant Structures

According to the specific application, structures and features with specific torsion resistant features have been designed and are reported in Table 1. Many of these structures have been commonly applied since the 1970s, as in the case of ship torsion boxes and torsional energy absorption devices, others have been introduced in the late nineties such as in the case of composite transmission power shafts. Some, such as the adaptive torsion wings, have only been conceptually modeled and lately investigated.

It can be observed that the common-designed torsion-resistant structures are characterized by some limitations. For instance, many of them are quite complex from the geometrical point of view: on one hand, this leads to the high complexity of numerical stress analysis and can affect the stress distribution, eventually causing local stress concentrations. On the other hand, the complexity of structures increases the costs of design and production of up to more than 250% if compared to conventional structures. The other main issue is weight increase, which could affect the performance of the components.

It is important to highlight the necessity to overcome these evident limitations through a transversal approach, considering the impressive power of nature-designed solutions [74].

## 3. Overcoming the Limitations of Traditional Structures from a Natural Perspective

### 3.1. Nature as a Source of Inspiration

**Figure 3:**
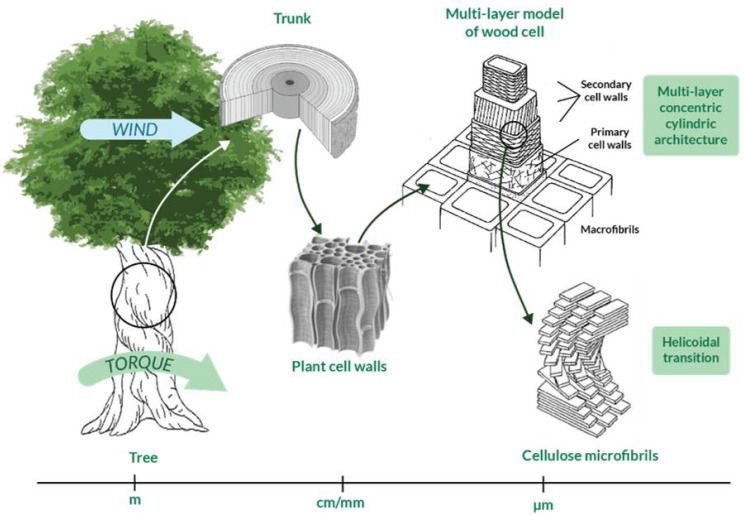
Hierarchical structure of wood from macroscale to microscale. Multi-layer concentric cylindric architecture and helicoidal transition are peculiarities that allow for torsion resistance and a progressive transition of properties, respectively.

Innovative solutions to face the torsional issue have recently been searched in nature, interrogated as a source of inspiration [74,93]. Many natural structures are subjected to torsional loads; some examples are tree trunks and wood cells and every bird and insect wing. Nature deals with torsion since the first birth species and the result is that there exist many systems in nature that develop torsion resistance through specific mechanisms and/or structural arrangements. For this reason, researchers consider nature as a qualified source of inspiration to develop torsion-resistant structures. Indeed, biomimicry and bio-inspiration are sciences based exactly on this concept [94], according to which scientists should respectively mime nature or let their research be inspired by it, lowering as much as possible the impact on the Earth and obtaining more sophisticated technologies, processes, and ecosystems [95,96]. Biological materials are able to optimally perform under different loads due to their complex and hierarchical structures, which go from macroscale to microscale [97]. Indeed, biological structures vary at different levels [98] and the interaction between them could provide specific torsional properties to the system as a whole. As an example, the complex hierarchical structure of wood is illustrated in Figure 3 and is characterized by more than five levels of hierarchy, as described in International Standard ISO 18457 (2016) on biomimetics [99,100]. Note that the multi-layer concentric cylindric structure of wood cells provide torsion resistance [101] and that the helicoidal transitions at the microscale avoid discontinuities in the change of properties between different levels of the entire structure [102].

As explained in depth by Huang et al. [103], the analysis of biological materials is quite intricate from the point of view of the computational analysis, also considering that some properties could derive from the interaction between structures at different levels of hierarchy [104]. To shed some light on this complexity, a characterization through multi-scale computational models can be taken into consideration [105,106].

### 3.2. Torsional Load in Nature: The Need of a Specific Problem Definition

**Figure 4:**
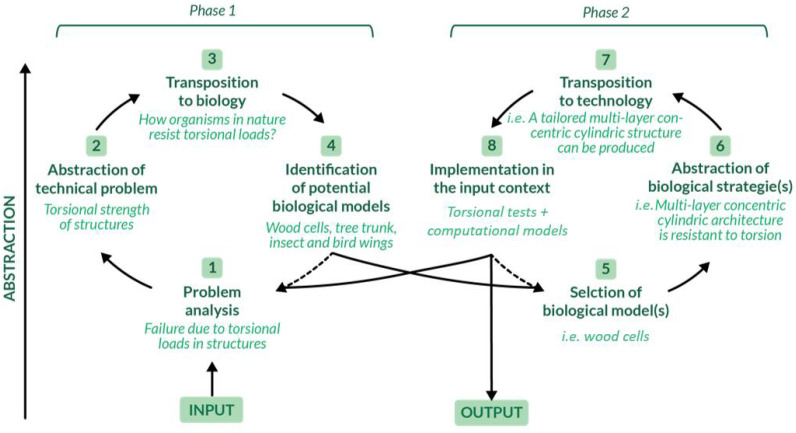
A schematization of the process model of biomimetics to address the torsional issue is presented. It is divided into two phases and eight steps: each of these steps takes advantage from specific tools such as problem analysis, abstraction, transposition, etc. [99,108]. Adapted with permission from [99].

As mentioned in Section 3.1, in order to overcome the limitations identified in traditional-designed torsion resistant structures, an interesting key could be bio-inspiration. However, to find an optimized solution to the torsion complex issue, a methodical approach should first be implemented. Indeed, natural structures that proved to have specific properties and functionalities could be investigated through characterization at various levels of the hierarchical structure, allowing the property of interest to be isolated [107]. Process models to approach bioinspired research have been proposed and analyzed, as also pointed out by Fayemi et al. [99] and Katiyar et al. [94], who suggested a unified problem-driven process (Figure 4). Taking into consideration different levels of abstraction, the problem can be divided in two phases: for each of them there are four steps that allow the biomimetic complex issue to be solved.

To develop torsional bioinspired structures, the problem analysis should be performed first (i.e., the description of the problem related to torsion resistance). Examples of problem analysis are reported in Section 1.1, together with a detailed collection of components and structures commonly subjected to torsional damage or failure. There are many fields of interest in which the development of an optimal torsion resistant structure might lead to an improvement in conventionally designed engineering components. For this reason, potentially prominent biological models were analyzed and critically compared.

### 3.3. Torsion-Resistant Nature-Inspired Structures: Biomimetic Unit Cells

In order to develop bioinspired innovative structures and prevent failures and damage due to torsional loading, some potential unit cells and geometries of biological structures were considered for the analysis and are schematized in Table 2, based on the screening in the abstraction tool [99]. First, some peculiar structures are present in ivory, a highly non-isotropic material with complex three-dimensional structures. Indeed, in every tusk, a core of dentine, usually referred to as ivory, is present: it is made of a matrix of micrometric cuboid [109] particles in a ground substance that contains dentinal tubules. These are cylinders aligned in sheets forming micro laminae, which are generally axially oriented, but could even be radially disposed, angled to the forming face or wrapped to form a helix [110]. Note that dentinal tubules might be curled into waves or could be straight and within micro laminae, they can be radial and angled to the axis (Table 2, first unit cell), specifically varying torsion-resistant properties. Finally, dentinal tubules may have the same orientation in adjacent micro-laminae, or orientation may change in the presence of a helicoidal pattern, obtaining a multi-layer concentric cylindrical architecture that is also present in the osteons of bones [111]. Considering that every kind of ivory has evolved its own structure to answer different needs such as increased strength and toughness [109], optimized bending and torsion resistant structure could be inspired by ivory. Many types of tusks could be investigated, as undertaken by Locke [109], who showed the macroscopical features of tusks. In the same study, microscopical details of different species of ivory such as dentine tubule arrangement in micro laminae, were identified. Focusing on the structure of narwhal tusk, a macroscopic life-handed helix spiral could be recognized [112]: it is characterized by an angle of inclination of the spiral arc of 66.88 ± 0.61° [113]. Mechanical properties of narwhal tusk have been examined by some studies [13,114], which established that tusk dentine is not a homogeneous material and is characterized by anisotropic properties. No specific studies related to the torsional test of narwhal tusk have been conducted, but an analogous helicoidal structure has been tested under torsion, exhibiting prominent behavior as in the case of a helix-reinforced composite, as reported by Porter et al. [86].

Another inspiring solid structure is the multi-layer concentric cylindrical cell wall architecture of plants [101] and bone osteons [115,116,117] (Table 2, second unit cell): stiff helicoidal micro-fibers are arranged parallel to each other in every microfibril thick lamellae [14,102,118], each one oriented according to a specific direction with respect to the cell axis. That direction is referred to as the microfibril or winding angle, in the range of 0 ± 90° [101], and it is the angle between a lamella and the subsequent one. This structure allows wood and bones to obtain superior mechanical properties to both bending and torsion through the optimization of the plies and angles combination [117]. Helicoidal layers of fibers arranged according to a multi-layer concentric cylindrical architecture are widely diffused in other recently investigated biological materials such as in insect cuticle and skeleton of glass sponges [119]. It is interesting to point out that helicoidal cell walls might be characterized by a far more complex texture: it might be formed by a mechanism based on geometrical considerations, which witnesses that the cell is equipped with intrinsic tools to generate a large variety of load-bearing textures [118]. Macroscopic plant structures are also characterized by helicoidal features; these structures increase torsion resistance, which is useful when wind forces are present. Indeed, in Figure 3, the mechanism according to which the wind can cause torsional load on a tree [120] is schematized; note that stem, roots, and soil altogether resist the generated torque. In fact, as explained by Skatter in his study [121], trees are mainly subjected to torsion due to wind forces, especially when they have asymmetric crowns: few decimeters of asymmetry can cause shear failure of the stem.

To avoid this, it has been demonstrated that spiral grain (Table 2, third unit cell) in the direction of wind-induced torque increases the bending and torsion strength of the stem and thus a beneficial configuration is obtained [121,122]. As a matter of fact, a spiral grain stem bends and twists more than a straight-grain stem when exposed to strong wind: through this mechanism of deformation, it offers less wind resistance and is less likely to break. A similar multi-layer architecture could be found in reinforcement geodetics [123], which are geometries on curved surfaces given by geodesic lines of which there are four kinds: the annular model, the single helicoidal thickening model, the double helicoidal thickening model, and the straight lines parallel and perpendicular to the axis mode. In the annular model, a series of annuli is arranged in parallel planes and is perpendicular to the axis of the cylinder, decreasing the buckling possibility. In nature, this reinforcement type is present in both plant-cells [123] and bird bones [90], where annuli are known as ridges, that, however, do not have a disruptive impact on the torsion-resistance of the entire structure. In the single helicoidal thickening model, thickenings have the function of strengthening the biological walls and preventing their collapse. For example, helical cell-wall thickenings observed in the root cortex cells of many Asplenium species mechanically stabilize the cortex tissue [124]. Helicoidal structures are commonly found in the peripheral body locations of plants and animals to prevent surface failure: indeed, if the beam is bent or twisted, the greatest stresses are concentrated on the surface, so an external strengthening mechanism could prevent failure. Furthermore, it has been well established that helicoidal components act as shearing force protection, that is, a specific point of interest for the purpose of this review [102]. One proof of this is the helicoidal arrangement of tension-resisting fibers observed in the stem of young herbaceous plants such as sunflowers. They provide wrapping for flatworms and roundworms: their outer membrane might be characterized by two different fiber arrangements. Fibers might run lengthwise and circumferentially or run helically, with left and right-hand helices, determining utterly different responses to the various torsional stresses the structures might encounter, as described by Vogel [125] and Neville [102]. Specifically, the helically reinforced model smoothly deforms responding to both tension and compression, but strongly resist torsional stresses: according to Vogel [125], the sets of left and right-handed helically arranged fibers resist twist in all directions. The value of deformation and torsional strength depends on the material that characterizes the structure. A fundamental parameter for helically reinforced surface membranes, and more in general helical torsion-resistant systems, is the “fiber angle”. It is the angle forming between the fibers and the long axis of the cylinder, and explains the relationship between the structure and its mechanical behavior, often derived with the aid of computational models [51,86,101,114,116,121,126,127,128].

A different example of a natural torsion-resistant unit cell in which helices are present is Bouligand’s structure [129] (Table 2, fourth unit cell), largely diffused in most arthropod cuticle. Indeed, the arthropod epidermal cells are characterized by a periodic architecture, with a helicoidal stacking of unidirectional chitin–protein fibrils set in an amorphous matrix [102,130]. The name of this structure comes from Bouligand, who dedicated his studies on the description of this twisted fibrous arrangement, which has then been largely observed in biological materials and tested under torsion [129]. Typically, Bouligand’s model could be recognized by a characteristic parabolic pattern that can be geometrically interpreted as an oblique section visualization of the layered and twisted structure resembling plywood [131], where consecutive layers of fibrils have a constant angle of twisting.

Bouligand’s structure has been proven to have a remarkable fracture toughness [140], well beyond its constituents, thanks to a combination of two main propagation modes controlled by that arrangement of chitin–protein: crack twisting and bridging (Table 2). Indeed, before the fracture begins, the twisted plywood allows reorientation and deformation of fibers in response to torsional loadings [144]. In other words, Bouligand’s structure ductility and toughness are biologically designed to prevent fracture through changes in the structural arrangement. Furthermore, Bouligand’s structure has exceptional stiffness and hardness [143,145,146] and, similar to that discussed for ivory, optimized bending and torsion resistance can be inspired by this structure, as proven by works such as the one by Nikolov et al. on high-performance composite structures [141]. To provide an idea of the wide diffusion of this architecture in nature, it is worth mentioning that arthropods are a kind of invertebrate animal that covers more than half the classified species [138], proving that their biological structures, and specifically their torsion-resistant properties, are highly performing and adaptable [129,130,139,140,141,142,143,147,148,149]. Arthropods include insects, arachnids, myriapods, and crustaceans that have a cuticle with a twisted plywood. Furthermore, Bouligand’s structure can be associated with micro- and nanoscale architecture (i.e., cholesteric liquid crystal), which, as explained by Mitov [150], are omnipresent in nature: chitin, cellulose, collagen, and silk are characterized by the Bouligand arrangement [102].

## 4. From Nature to Novel Materials: Torsion-Resistant Metamaterials

Eventually, nature-inspired structures have been exploited in the design of novel metamaterials that are able to actively address the torsion-resistance issue with innovative and customized solutions. Following the problem-driven approach defined in Section 3.2, phase 2 is now faced and the biological strategies abstracted from natural torsion-resistant unit cells are transposed to technology and tested.

**Figure 5:**
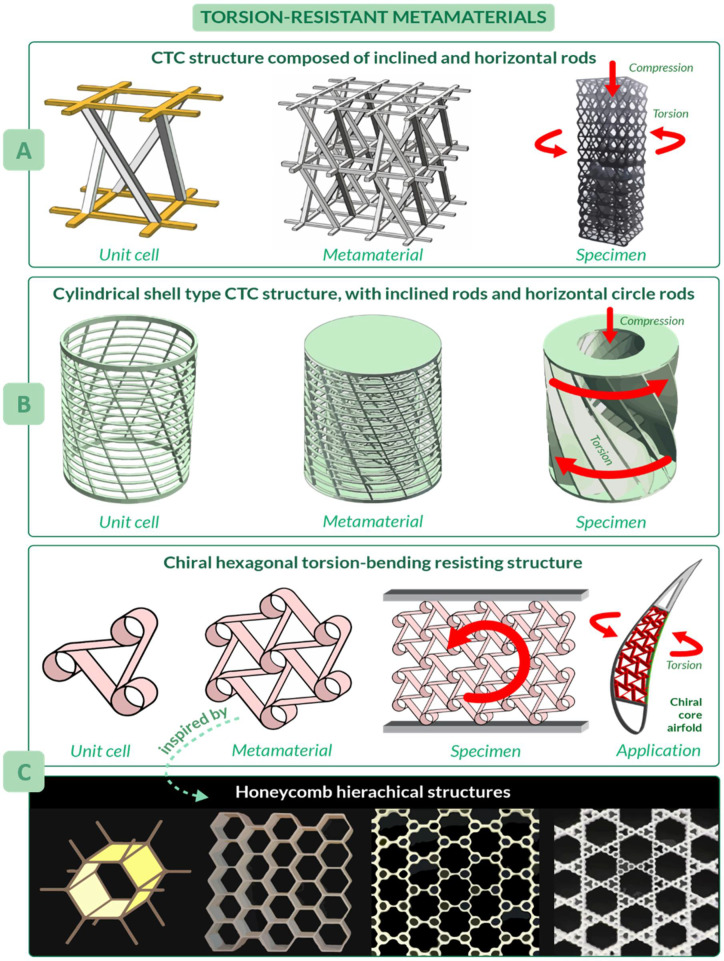
Torsion-resistant metamaterials. (**A**) Compression–torsion–conversion (CTC) structure composed of inclined and horizontal rods. (**B**) Cylindrical shell type CTC structure. (**C**) Chiral hexagon torsion-bending resistant structure.

Metamaterials are characterized by properties not simply given by their composition, but arising from their structure [34]; they are usually assembled starting from one or more basic unit elements that repeat themselves, forming a clinical pattern [151]. An example is the metamaterial conceived by Zhong et al. [33], which was able to convert axial compression (or tension) into torsion: the unit cell, the following metamaterial, and the final tested specimen are illustrated in Figure 5A. Note that the unit cell of this material is characterized by an arrangement of rods, which resembles the helical structures described in Section 3.3. The proposed design allows for a promising peak value of 16.2° of torsion angle to be reached. Possible applications of this metamaterial are wave converters, able to transform shear waves into longitudinal waves and vice versa, or morphing structures in aircraft and aerospace engineering [33].

Another compression-torsion-conversion (CTC) metamaterial has been proposed by Wang et al. [32], which focused their attention not only on the properties of the final structure, but also on the main problem of metamaterials such as inefficient use of space. Their work introduces a cylindrical metamaterial, increasing the capability of compression and torsion resistance [32]. Its unit cell has inclined and horizontal circle rods, which resembles the helical and annular features of natural unit cells, respectively (Figure 5B). The most crucial benefit of this structure is the tailored torsion resistance coming from the relationship between rod inclination angle and the torsion angle of the rotation spring: the larger the rod inclination angle and slenderness ratio, the larger the torsion angle. The metamaterial consists of three of the cylindrical shells, differing in radius, arranged one inside the other. Since manufacturing of these structures, also known as rotation springs, is quite problematic, a continuous structure with curved surfaces has been proposed for tests. Applications of these metamaterials include structures of machinery and vehicles.

Considering the need to design lightweight components, promising metamaterials have been inspired by honeycombs. As shown by Haghpanah et al. [152], from the basic concept of hexagonal honeycomb, more complex hierarchical structures with an improved efficiency could be developed, as in the case of self-similar hierarchical honeycombs shown in Figure 5C. An example of structure inspired by honeycombs is the morphing airfoil designed by Bettini et al. [153,154], where a composite chiral element, which resembles honeycomb hexagons and spirals (largely diffused in nature [123,155,156,157]), was used in the core of the airfoil to resist torsion and bending during flight. To be more precise, this metamaterial can improve morphing performances by increasing the maximum allowable displacement, which can reach in tension up to 12% of cell dimension and about 30% in compression.

## 5. Conclusions and Future Perspectives

Failure of components due to torsional load is an impactful issue that has been addressed across the years, starting from a simplified analytical approach and reaching time-demanding computational simulations. However, the complexity of the problem, its three-dimensional nature, the combination of torsional load with other kinds of stresses (i.e., compression, bending or tension) make its systematic analysis particularly hard.

In order to overcome these limitations, a methodical approach was proposed in this review, starting from a punctual analysis of the problem. Several torsional failures have been deeply investigated and categorized in different fields of interest. Common-designed solutions to increase torsional resistance are critically compared and their drawbacks (i.e., high complexity and weight) are the preparatory point for a deeper nature-driven analysis.

For this reason, nature is considered as a mine of disruptively novel ideas: helicoidal laminae cylinders, multi-layer concentric cylindric architectures, helically externally reinforced cylinders, cylinders with external helical grains, and Bouligand’s structures have been identified as prominent candidates to address the torsional problem. These architectures are commonly found in both plants and animals, which should resist torsional load during their life-cycle without deteriorating their mechanical characteristics.

After this precise screening, a transposition to technology was proposed: biological strategies commonly implied by torsion-resistant bio-inspired unit cells are exploited in novel metamaterials, which present a multi-scale specific arrangement to address the torsional issue. Their properties do not come solely from the characteristics of the base materials, but from their newly arranged structure. Their precise torsion-resistant bio-inspired shape, combined with multi-scale architecture and tailored orientation of the inner fibers make them future prominent candidates to effectively address the design drawbacks encountered in traditional torsion-resistant structures.

## Figures and Tables

**Table 1:** Common-designed torsion resistant structures. In the table, the fields of interest, a schematic of torsion-resistant components, their potentialities, and limitations are highlighted.

Structure	Field of Interest	Schematic of the Structure	Potentialities	Limitations
Torsional energy absorbing devices [36]	Civil engineering	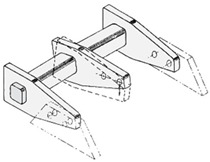 Adapted with permission from [36]. Copyright 1972 J. M. Kelly, R. I. Skinner, A. J. Heine	Sole specific task to absorb kinetic energy generated in the structure; Independent device with respect to the structure as a whole; Allow the structure to operate under simpler and less severe conditions: better distribution of deformation.	High costs; Torsional load in cyclic conditions are entirely sustained by this device [36].
AR-Brace energy absorbing devices [22]	Civil engineering	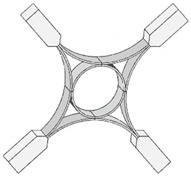 Adapted with permission from [22].	Lower inelastic energy dissipation on the structure’s framing system, reducing structural damage; Reduce floor accelerations and base shear; Reduce structural torsion adding both rigidity and dumping.	High complexity of the device, which is intended for passive control of vibrations and vibration-dependent responses.
Helicoidal steel reinforcements in concrete [7,84]	Civil engineering	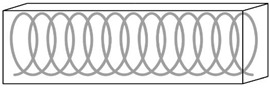	Overcome the limitations in space and strength; If the helical reinforcement varies between 0.4 and 1.0%, torsional strength increases from 20 to 50%, regardless the fact that of longitudinal bars are added or not [7,84]; Increase reinforced beam ductility (at least 400–600% more deflection).	Advantages are observed only if a high compressive strength concrete (70 MPa) is used instead of common concrete (32 MPa) [7,84]; Adjustments do not respect norms requirements (AS3600); +250% increase in costs [85].
Composite power shafts [47,51,52,85,86,87,88,89]	Mechanical engineering	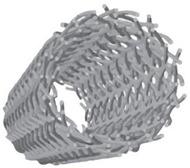 Adapted with permission from [51].	Increase in torque capability of 160% with respect to hollow conventional shafts; Mass reduction of 75% with respect to hollow conventional shafts; Elastic properties can be tailored to increase torque and rotational speed.	Stress intensity factors at crack tip and holes must be studied for inhomogeneous materials.
Wings internal struts [90]	Aircraft engineering	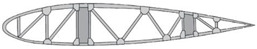 Adapted with permission from [90].	Increase in torsional stiffness, up to 7 times the open cell foam structure; Lightweight structure; Limited deformability.	Complexity of stress analysis in frame structures; Struts must be placed exactly where combined torsion and bending are most dangerous.
Active aeroelastic structure devices [91]	Aircraft engineering	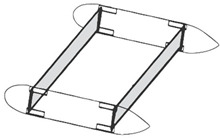	Torsional stiffness can be reduced while limiting shear center shift through translation of both front and rear web inwards.	Very high complexity in the design and control; Increase in system weight (+2–5% of the structural wing weight) [91].
Torsion boxes [74,92]	Marine engineering	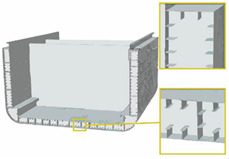 Adapted with permission from [92].	Lower stresses generated in the hull thanks to: large torsional stiffness in the cellular configuration; reduction of huge stress concentrations caused by axial or shear stresses in torsion	Introduction of geometric discontinuities in the hull of ships; Complex torsional and flexural loads characterization.

**Table 2:** Torsion-resistant biomimetic unit cells are selected, the biological source, and the schematic of the structure is reported. Additionally, specific torsion-resistant features are highlighted, in accordance with the performed mechanical/numerical tests.

Bio-Inspired Structure	Biological Organism	Unit Cell Structure	Torsion Resistant Features	Performed Tests
Helicoidal laminae in solid or hollow cylinders [86,110,112,114,128,132]	Tusk of narwhal, hippopotamus, African and Indian elephant, sperm and killer whale, boar, walrus	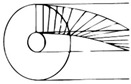 Adapted with permission from [110]	Fibers tangential to cylinder’s axis are helicoidally arranged.	Helix-reinforced composite, ZrO_2_ and epoxy (60:40), 45°: Shear strength: 5.5 ± 0.7 GPa Ivory: Flexural strength: 378 MPa Fracture toughness: 2 MPa m^1/2^
Multi-layer concentric cylindric architecture [100,101,115,118,133,134]	Wood cells, bone osteons, insect cuticle and skeleton of glass sponges	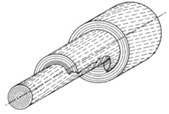 Adapted with permission from [14]	Cylindric layers can have: aligned fibers in each layer with cylindrical helicoidal grading; helical fibers with variable angles of pitch with a more complex texture.	0Wood cell-wall with cellulose microfibril angle of 50° → fracture strain: 13.5%; Bone osteons → compressive modulus of lamellae: 20 GPa Wood-inspired composite → compressive modulus variation for a winding angle of 45°: +150%
Helically reinforced cylinder [14,90,102,121,122,124,125,135,136]	Root cortex cells of most Asplenium species, herbaceous plants (sunflower), tree stem	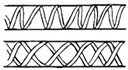	Fibers arranged according to single or double helices on a cylindrical surface.	Tree stem (14 cm of diameter) → breaking load in torsion test: 275 MPa
Twisted plywood (Bouligand’s structure) [102,129,130,131,134,137,138,139,140,141,142,143]	Arthropod cuticle (crab, lobster, mantis shrimp, arachnids and myriapods),	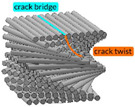 Adapted with permission from [143]	Layered and twisted structure in which consecutive layers of parallel fibers have a constant angle of twisting.	Bouligand composite structure, RGD720 and polyester (22:78) → Peak torque: 7.5 Nm, Rotation: 2.0 ± 5.3 × 10^–2^ rad Impact forces repetitively endured: ≤1500 N

## Data Availability

Not applicable.
